# A Mechanism for Synergy with Combined mTOR and PI3 Kinase Inhibitors

**DOI:** 10.1371/journal.pone.0026343

**Published:** 2011-10-19

**Authors:** Shujie Yang, Xue Xiao, Xiangbing Meng, Kimberly K. Leslie

**Affiliations:** 1 Department of Obstetrics & Gynecology, The University of Iowa, Iowa City, Iowa, United States of America; 2 Holden Comprehensive Cancer Center, The University of Iowa, Iowa City, Iowa, United States of America; National Cancer Institute, United States of America

## Abstract

Dysregulation of the mammalian target of rapamycin (mTOR) signaling has been found in many human cancers, particularly those with loss of the tumor suppressor PTEN. However, mTORC1 inhibitors such as temsirolimus have only modest activity when used alone and may induce acquired resistance by activating upstream mTORC2 and Akt. Other tumors that do not depend upon PI3K/Akt/mTOR signaling for survival are primarily resistant. This study tested the hypothesis that the limited clinical efficacy of temsirolimus is due to a compensatory increase in survival signaling pathways downstream of Akt as well as an incomplete block of 4E-BP1-controlled proliferative processes downstream of mTOR. We explored the addition of a PI3K inhibitor to temsirolimus and identified the mechanism of combinatorial synergy. Proliferation assays revealed that BEZ235 (dual PI3K/mTOR inhibitor) or ZSTK474 (pan PI3K inhibitor) combined with temsirolimus synergistically inhibited cell growth compared to cells treated with any of the agents alone. Co-treatment resulted in G0/G1 cell cycle arrest and up-regulation of p27. Cell death occurred through massive autophagy and subsequent apoptosis. While molecular profiling revealed that, in most cases, sensitivity to temsirolimus alone was most marked in cells with high basal phospho-Akt resulting from PTEN inactivation, combining a PI3K inhibitor with temsirolimus prevented compensatory Akt phosphorylation and synergistically enhanced cell death regardless of PTEN status. Another molecular correlate of synergy was the finding that temsirolimus treatment alone blocks downstream S6 kinase signaling, but not 4E-BP1. Adding BEZ235 completely abrogated 4E-BP1 phosphorylation. We conclude that the addition of a PI3K inhibitor overcomes cellular resistance to mTORC1 inhibitors regardless of PTEN status, and thus substantially expands the molecular phenotype of tumors likely to respond.

## Introduction

Alterations in the phosphoinositide-3-kinase (PI3K)/Akt/mammalian target of rapamycin (mTOR) signaling pathway have been found in many human tumors. In particular, amplification and mutation of *PIK3CA*, mutation of *PIK3R* and Akt, and loss of tumor suppressor PTEN (phosphatase and tensin homolog deleted from chromosome 10) contribute to constitutive activation of this signaling pathway [1], [2], [3], [4]. Understanding the interplay among signaling molecules in the PI3K/Akt/mTOR pathway is of utmost importance. Two distinct mTOR complexes, mTORC1 and mTORC2, have been identified and have differential sensitivity to rapamycin. mTORC1 is downstream of Akt, sensitive to rapamycin inhibition, and controls cap-dependent protein translation [5]. The two best-studied mTORC1 substrates are 40S ribosomal S6 kinase 1 (S6K1) and eukaryotic translation initiation factor 4E-binding protein 1 (4E-BP1), which mediate efficient protein translation. In contrast, mTORC2 is directly upstream of Akt and is resistant to rapamycin. Akt can be activated by phosphorylation at two different sites, S473 by mTORC2 and T308 by phosphoinositide-dependent kinase 1 (PDK1). Constitutive activation of the PI3K/Akt/mTOR signaling axis leads to uncontrolled tumor cell proliferation and survival [1].

Given the importance of the mTOR pathway in cancer cell growth, significant efforts have attempted to identify targeted inhibitors. Rapamycin and its analogs (rapalogs), such as RAD001 (everolimus), AP23573 (ridaforolimus) and CCI-779 (temsirolimus) are allosteric inhibitors of mTOR [6]. However, single agent rapalogs have only achieved modest antitumor activity in the clinic [7]. The limited anticancer efficacy of the rapalogs can be explained by two possible mechanisms: (1) rapalogs inhibit only mTORC1 (not mTORC2), thereby inducing feedback activation of survival signaling pathways such as Akt phosphorylation [7], [8], [9]; or (2) rapalogs incompletely block mTORC1 downstream signaling. For example, in some cells mTOR inhibitors prevent phosphorylation of S6K1 but not 4E-BP1, thus allowing the cells to escape growth inhibition [10], [11], [12].

Previous studies indicate that PTEN inactivation, *PIK3CA* mutation, and mTOR dysregulation are common molecular signatures for endometrial carcinoma [1], [13]. Furthermore, PI3K activation is a hallmark for aggressive tumors at this site [14]. mTOR inhibitors (temsirolimus, everolimus, and ridaforolimus) have been tested in phase I and II clinical trials for advanced and recurrent endometrial carcinomas with some promising clinical outcomes; however, response rates are not robust. In general, responses are partial and vary from 8%–26% with an additional 20%–63% of patients achieving stable disease for at least four months [15]. Some patients achieve no benefit from therapy (primary resistance), whereas in others, stable disease or an initial response occurs. Nevertheless, most patients eventually experience progression of disease (acquired resistance). More information will be available following the analysis of the phase II trial of temsirolimus for advanced endometrial cancer, Gynecologic Oncology Group trial 248; however, since this trial only recently closed to accrual, the outcome data are not mature.

In this current study, we investigated how inhibition of mTOR can be optimized. We examined the growth inhibitory effect of temsirolimus on a panel of endometrial cancer cells and observed differential sensitivity as well as compensatory Akt phosphorylation in a subset of cell lines, which may represent one mechanism for acquired resistance. We identified cells which were primarily resistant to treatment and compared these to other cells which initially responded but employed escape mechanisms to achieve acquired resistance. To overcome both forms of resistance, we applied dual inhibition of PI3K and mTOR to prevent cell survival signaling. Our data reveal that combination treatment of temsirolimus with either BEZ235, a dual PI3K/mTOR inhibitor, or ZSTK474, a pan PI3K inhibitor, blocked Akt activation and inhibited phosphorylation of both 4E-BP1 and the substrate for S6K, ribosomal S6 (rS6), which ultimately resulted in synergistic cell death.

## Materials and Methods

### Cell lines

Six endometrial cancer cell lines (AN3CA, RL95-2, Hec1A, SKUT1B, ECC-1, and KLE) were obtained from ATCC and grown according to the recommended guidelines. Two endometrial cancer cell lines, Ishikawa H and Hec50co (gifts from Dr. Erlio Gurpide, New York University), were grown in DMEM supplemented with 10% fetal bovine serum (FBS) and penicillin-streptomycin, all from Gibco.

### Reagents

Temsirolimus, NVP-BEZ235, and ZSTK474 were purchased from LC Laboratory and resuspended in DMSO. Antibodies against Akt (#4691), Akt P-S473 (#4060), Akt P-T308 (#2965), rS6 (#2217), rS6 P-S235/S236 (#4858), p70S6K (#9202), p70S6K P-T389 (#9205), p27 (#3686), LC3 (#2775), 4E-BP1 (#9644), 4E-BP1 P-T37/T46 (#2855), and PARP (#9542) were from Cell Signaling. The PTEN (sc-7974) antibody was from Santa Cruz Biotechnology. The β-actin antibody (#A1978) was from Sigma Aldrich.

### Cell viability assays

Ninety-six-well plates were seeded with 10,000–20,000 cells in each well 24 hrs prior to drug treatment. Cells were treated with inhibitors or DMSO control for 3 days. Each experiment was carried out in triplicate. Effects on cell proliferation and loss of cell viability were evaluated with WST-1 (Clontech) assay per manufacturer's instructions.

### Western blotting

Equal numbers of cells were plated 24 hrs prior to treatment. Unless otherwise noted, cells were grown in the presence of inhibitors for 24 hrs. Cells were solubilized in cold NP-40 cell lysis buffer (150 mM NaCl, 50 mM Tris/HCl, pH 7.4, 1% NP-40, and a protease and phosphatase inhibitor cocktail from Pierce). Lysates were analyzed by Western blotting with specific primary and HRP-conjugated secondary antibodies.

### Cell cycle analysis

Cells were grown for 24 hrs in normal growth media followed by drug treatment for an additional 24 or 72 hrs. Floating cells were collected with the media, and attached cells were collected by trypsinization. After centrifugation, the cell pellets were fixed in 70% cold ethanol, treated with RNAase A, stained with propidium iodide, and prepared for flow cytometry. FACScan (BD Bioscience) was used to detect the samples and ModFit was used to analyze the cell cycle alterations.

## Results

### Cells display varying responses to temsirolimus

**Figure 1:**
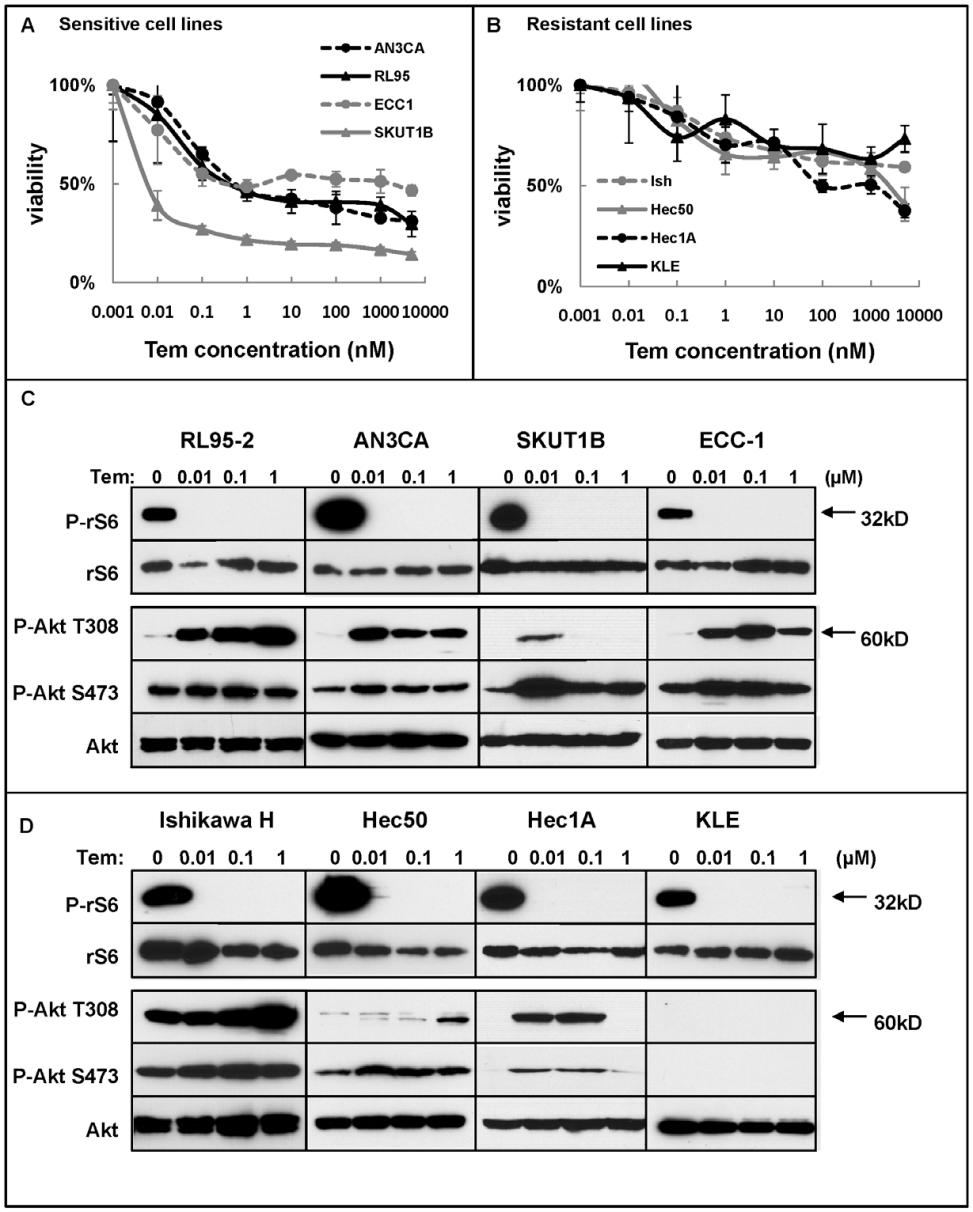
Temsirolimus differentially regulates cell viability and Akt phosphorylation in a dose-dependent manner. *A*, *B*, Endometrial cancer cells were treated with increasing doses of temsirolimus for 72 hrs. Results are separated by (A) sensitivity or (B) resistance as determined by cell viability. *C*, *D*, Phosphorylation of rS6 (P-rS6) and Akt (P-Akt T308 and P-Akt S473) after treatment with indicated doses of temsirolimus for 72 hrs in temsirolimus-sensitive (C) or temsirolimus-resistant (D) cells was determined by Western blotting. Expression of total rS6 and Akt protein serves as loading controls.

To establish a cell-based model system to understand the efficacy of mTOR inhibitors in endometrial cancer patients, we tested the growth-inhibitory properties of temsirolimus on eight endometrial cancer cell lines using *in vitro* proliferation assays. Proliferation of four endometrial cancer cell lines (SKUT1B, AN3CA, RL95-2, and ECC-1) was inhibited at low nanomolar concentrations of temsirolimus (Fig. 1A). The temsirolimus IC_50_ for these sensitive cells was approximately 1 nM (Supporting Table S1), indicating a strong growth inhibitory impact. In contrast, Ishikawa H, Hec50co, Hec1A and KLE cells were more resistant to treatment, and the average IC_50_, when reached, was at least 10-fold higher (Fig. 1B, Supporting Table S1).

To understand the signaling pathways responsible for the contrasting effects of temsirolimus on proliferation, we first confirmed functional inhibition of mTOR activity. Surprisingly, even in the sensitive cell lines, phosphorylation of mTOR at S2448 was only partially inhibited by temsirolimus (Supporting Fig. S1), suggesting that loss of mTOR phosphorylation does not distinguish between sensitive and resistant cells. However, temsirolimus completely prevented phosphorylation of rS6, which is downstream of mTORC1, in all the cells regardless of sensitivity and at all tested concentrations (Fig. 1C, D). This finding validates the functional inhibition of mTORC1 activity despite the persistence of some phosphorylation, although reduced, at the S2448 site (Fig. 1C, D and Supporting Fig. S1). We conclude that mTOR inhibition at S2448 and loss of rS6 activation as readouts do not adequately distinguish between cells that are sensitive initially versus those that are primarily resistant to temsirolimus-induced growth inhibition.

### Akt phosphorylation status distinguishes between sensitive and resistant cells

**Figure 2:**
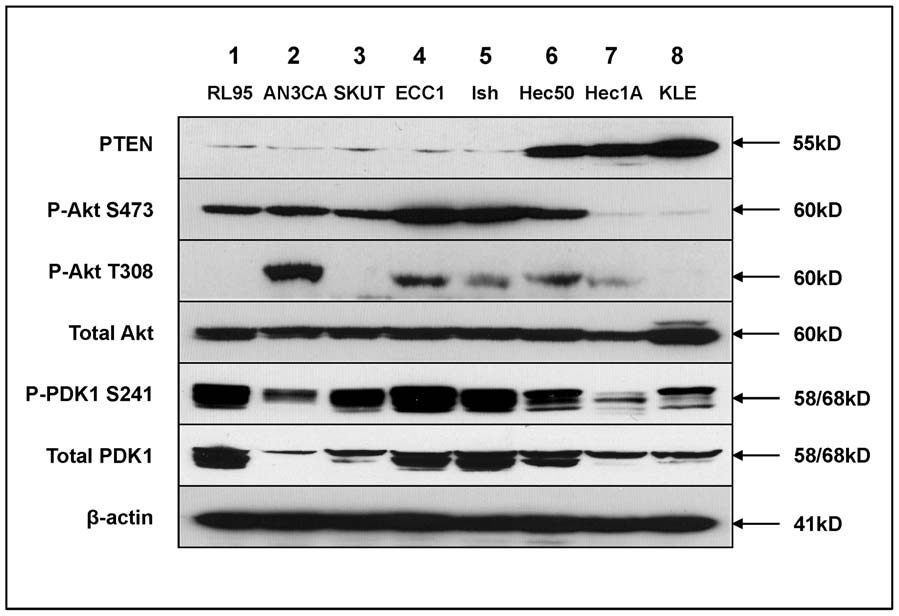
Baseline expression of PTEN, phospho-Akt and phospho-PDK1. Eight endometrial cancer cell lines were grown without treatment. Total protein extracts were analyzed by Western blotting for PTEN, phospho-Akt (P-Akt S473 and P-Akt T308), total Akt, phospho-PDK1 (P-PDK1 S241), and total PDK1. β-actin expression served as a loading control.

To understand the basis for cell sensitivity to temsirolimus as a single agent, we turned to an analysis of Akt activation, both at baseline and in response to treatment. We found that the baseline constitutive activation of Akt was predictive of cell sensitivity with the most resistant cells having very low basal S473 and T308 phosphorylation (Fig. 1C, D). After treatment with temsirolimus, a compensatory increase in Akt phosphorylation at both sites was detected in the most sensitive endometrial cancer cell lines tested (Fig. 1C), but the primarily resistant cells (KLE) demonstrated no Akt phosphorylation at either site, and another resistant line, Hec50co, showed reduced phosphorylation (Fig. 1D). Thus, in contrast to sensitive cells, primarily resistant cells have low basal Akt phosphorylation and do not respond with compensatory hyper-phosphorylation after temsirolimus treatment. Of note, phospho-PDK1, the kinase responsible for Akt phosphorylation at T308, is low in several resistant cell lines such as Hec1A and KLE (Fig. 2). This indicates primary cell resistance and a general lack of dependence on the Akt signaling pathway for proliferation. On the other hand, the finding of compensatory activation of Akt in responsive cells is consistent with previous reports in the literature of rapalog-induced Akt phosphorylation in many cancer cell lines, human xenograft models, and patient tumors [16]. Compensatory hyper-Akt phosphorylation suggests one potential mechanism whereby cells that are initially sensitive escape the growth inhibitory effects of the drug and become secondarily resistant. From these data, we distinguish between primary resistance with a lack of baseline Akt dependence versus acquired resistance demonstrated by hyper-Akt activation in response to temsirolimus.

### Baseline expression of PTEN as a correlate with Akt activation and primary sensitivity

Endometrial cancer is frequently accompanied by the loss of functional PTEN: 30%–83% have PTEN mutations or loss of PTEN expression [17], [18], [19]. Loss of the PTEN tumor suppressor has been proposed to correlate with mTOR inhibitor sensitivity in some other cancer cell lines and patient tumors [20], [21]. As discussed above, Figures 1 and 2 demonstrate the presence of basal phospho-Akt in some of the endometrial cancer cell lines, which we propose is a marker for initial sensitivity to single agent temsirolimus, with compensatory hyper-phosphorylation as a marker for developing acquired resistance. To understand the basis for high baseline Akt phosphorylation, we compared the baseline expression of PTEN and phospho-Akt in eight endometrial cancer cell lines. As shown in Figure 2, five cell lines (RL95-2, AN3CA, SKUT1B, ECC-1, and Ishikawa H) showed PTEN loss, and three cell lines (Hec50co, Hec1A, and KLE) expressed PTEN. For most cell lines, the exception being Hec50co, an inverse relationship was found between Akt phosphorylation at S473 and PTEN loss such that the cells with absent PTEN exhibited higher Akt S473 phosphorylation levels (Fig. 2). Strikingly, the cells with the lowest level of PTEN expression were also the most sensitive and responded with decreased cell proliferation when treated with temsirolimus (Fig. 1A). Conversely, the three cell lines with PTEN expression showed lower Akt S473 phosphorylation and were relatively resistant to mTOR inhibition. It should be noted that Ishikawa H cells have high Akt phosphorylation and loss of PTEN but are relatively resistant to temsirolimus. Hec50co cells, which have high Akt phosphorylation and maintain PTEN expression, are also resistant to temsirolimus. These data confirm the general, but not absolute, correlation between PTEN loss and constitutive Akt baseline activation at S473 as a marker for primary cell sensitivity in the most responsive cells.

### Dual therapy: PI3K inhibitors reverse temsirolimus-induced Akt phosphorylation

**Figure 3:**
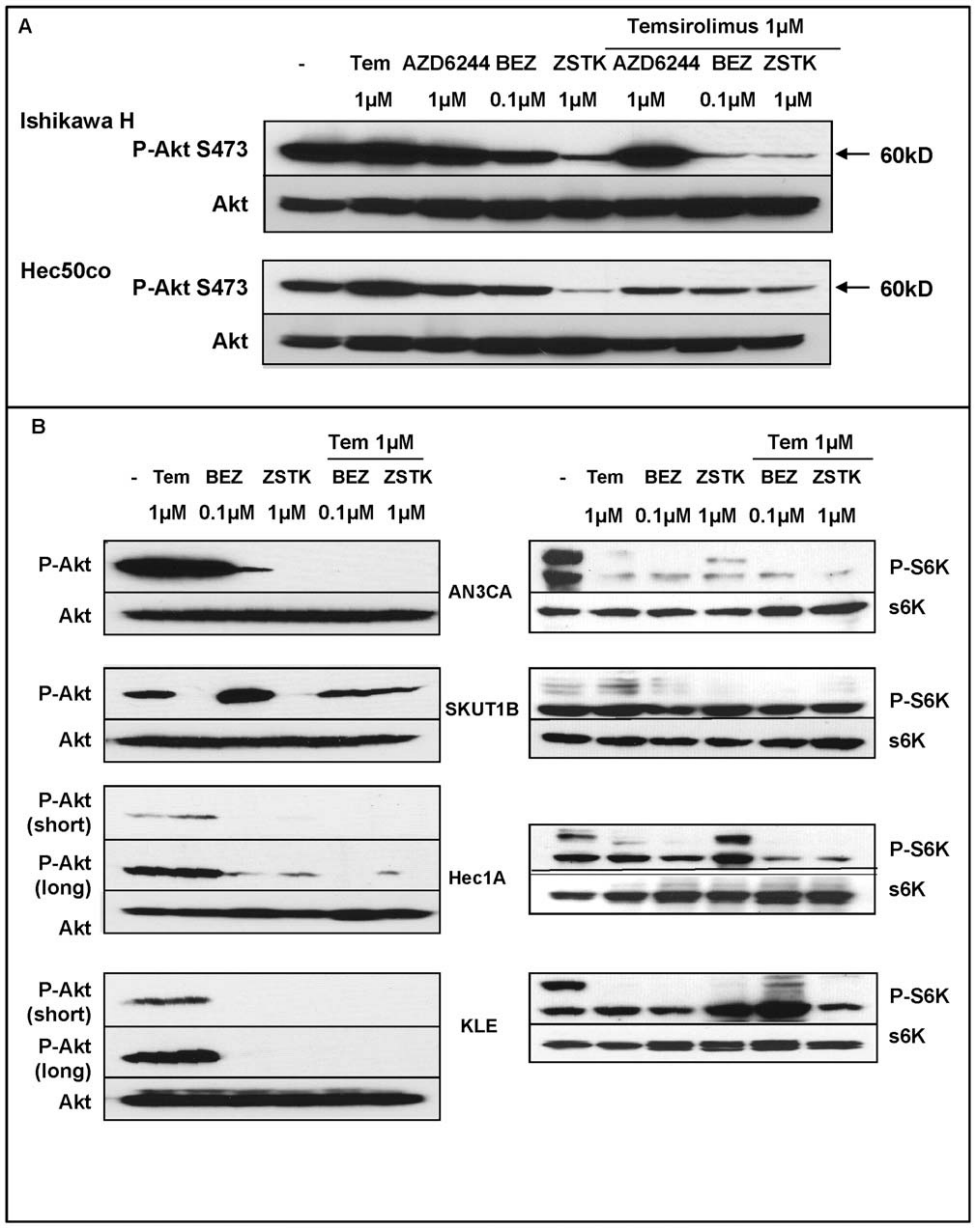
Temsirolimus-induced Akt phosphorylation is decreased by BEZ235 and ZSTK474, but not by AZD6244. *A*, Ishikawa H (upper panels) and Hec50co (lower panels) cells were grown for 24 hrs and treated overnight with the indicated inhibitors. Phospho-Akt (P-Akt S473) and total Akt were assessed by Western blotting. *B*, Endometrial cancer cell lines were treated with the indicated inhibitors overnight. Total protein extracts were analyzed by Western blotting for P-Akt S473 and total Akt (left panels) or phospho-p70S6K T389 (P-S6K) and total p70S6K (S6K, right panels). Phospho-Akt blots for Hec1A and KLE cells were subjected to a long exposure to visualize low levels of Akt phosphorylation.

To overcome the compensatory Akt phosphorylation induced by temsirolimus treatment as a mechanism of acquired cell resistance, six candidate inhibitors were chosen based on previously reported efficacy (Supporting Table S2). Ishikawa H (type I endometrial cancer cells) and Hec50co cells (type II endometrial cancer cells) [22] were first used to test the efficacy of these agents alone and in combination with temsirolimus. As shown in Figure 3A, Ishikawa H and Hec50co cells displayed a basal level of Akt phosphorylation that was further increased by temsirolimus treatment, with Ishikawa H cells showing higher levels compared with Hec50co cells, as discussed above (also, Fig. 1C, D). BEZ235 (dual mTOR/PI3K inhibitor) and ZSTK474 (PI3K inhibitor) decreased the basal level of Akt phosphorylation in both cell lines tested when used alone (Fig. 3A). Strikingly, when combined with temsirolimus, both BEZ235 and ZSTK474 blocked the temsirolimus-induced hyper-phosphorylation of Akt. We tested four other endometrial cancer cell lines and observed a similar pattern of inhibition of Akt phosphorylation (Fig. 3B, left panel). In order to demonstrate the effect of drug treatment even on resistant KLE and Hec1A cells, which have very low levels of basal phospho-Akt, we exposed the immunoblot for extended periods of time (Fig. 3B). The effect of the treatment regimens was preserved in these cells as well even though the baseline levels of phospho-Akt were substantially reduced. A marker for temsirolimus activity is the loss of phosphorylation of p70S6K, which is directly downstream of mTORC1. Accordingly, we detected a loss of phospho-S6K whereas total S6K levels were generally unchanged (Fig. 3B, right panel). This result confirms that whether temsirolimus is used alone or in combination, this cellular response is maintained.

In contrast to BEZ235 and ZSTK474, other molecular inhibitors including AZD6244 (MEK inhibitor), LBH589 (HDAC inhibitor), LY29004 (PI3K inhibitor), or AZD2171 (VEGFR/PDGFR inhibitor) did not reverse temsirolimus-mediated compensatory Akt phosphorylation in Hec50co and Ishikawa H cells (Fig. 3A and Supporting Fig. S2), though these inhibitors have been previously shown to block rapalog-induced Akt phosphorylation in other cell lines [23], [24], [25]. Based upon these data, we thus narrowed our focus to the study of BEZ235 and ZSTK474, which we proposed could overcome acquired resistance to mTOR inhibition.

### Combination treatment synergistically inhibits cell growth in endometrial cancer cell lines

**Figure 4:**
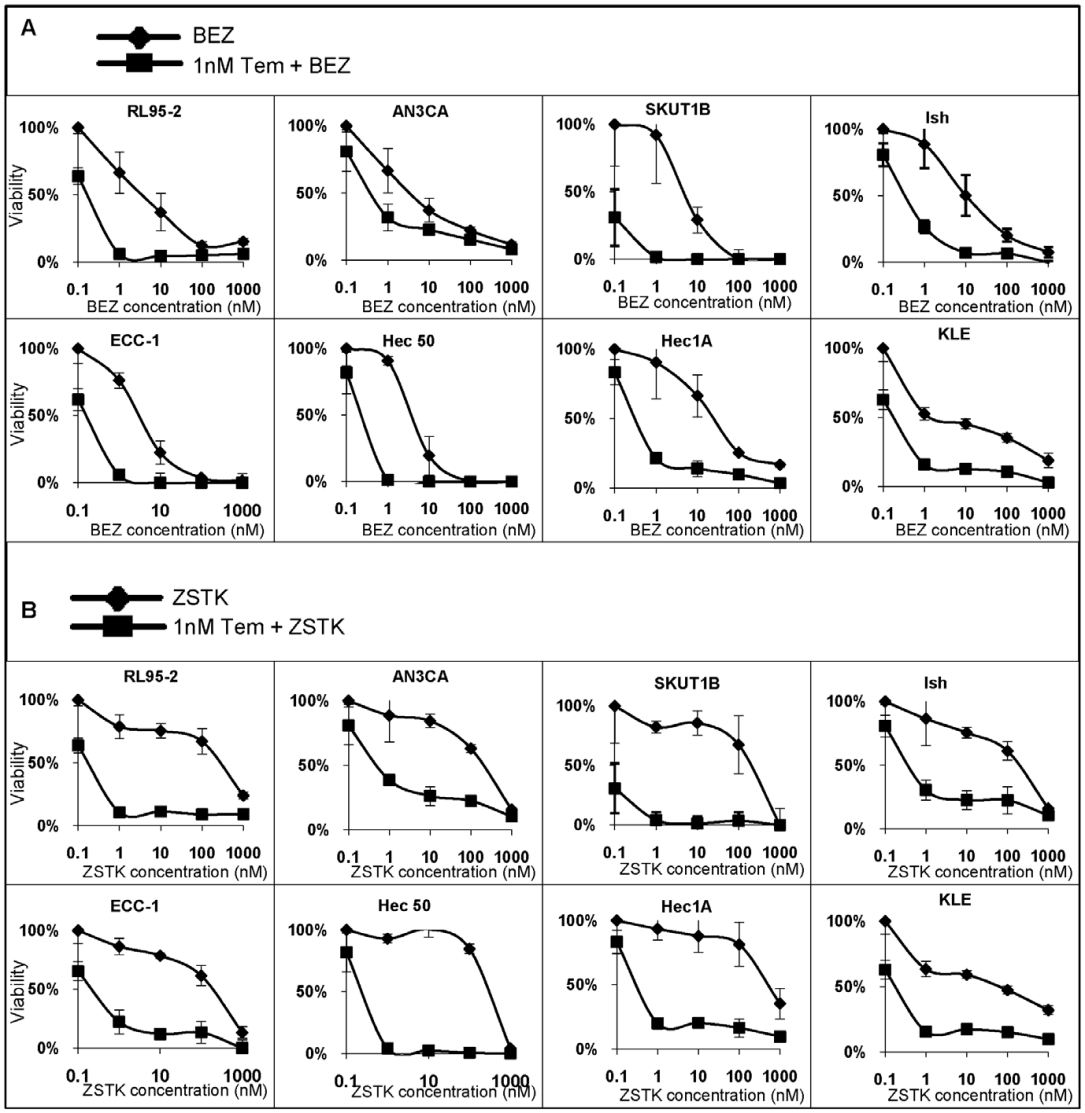
Combination treatment of BEZ235 or ZSTK474 with temsirolimus synergistically inhibits cell proliferation. *A*, *B*, Cell viability was determined in the indicated endometrial cancer cell lines after treatment with increasing concentrations of (A) BEZ235 or (B) ZSTK474 alone or in the presence of 1 nM temsirolimus for 72 hrs.

After demonstrating BEZ235 and ZSTK474's effectiveness in down-regulating temsirolimus-induced Akt hyper-phosphorylation, we examined the anti-proliferative properties of combined treatment of temsirolimus with BEZ235 or ZSTK474. A broad range of doses — either BEZ235 or ZSTK474 alone or in combination with temsirolimus— were tested in order to determine the optimal concentration for eliciting growth inhibitory effects. First, the panel of endometrial cancer cells were treated with single drug (BEZ235 or ZSTK474 at 1–1000 nM) and compared to vehicle (DMSO) control. BEZ235 alone reduced cell proliferation by 50% at doses as low as 1–50 nM (Fig. 4A). ZSTK474 alone was cytostatic in all the eight tested endometrial cancer cell lines; it inhibited cell growth at about 100–1000 nM (Fig. 4B).

However, when either BEZ235 or ZSTK474 was combined with low dose temsirolimus (1 nM), cell proliferation was inhibited in a synergistic manner compared to BEZ235 or ZSTK474 alone. Notably, a 50% reduction in proliferation occurred with combined temsirolimus and BEZ235 at a lower concentration of BEZ235 than with BEZ235 alone (Fig. 4A). Co-treatment of ZSTK474 with 1 nM temsirolimus resulted in a dose-dependent synergistic effect on cell proliferation at concentrations similar to BEZ235 with temsirolimus (Fig. 4B). The synergistic effect was seen in all cell lines except KLE, in which the drug combinations exhibited an additive effect. Combination indices were calculated and are shown in Supporting Table S1. These data signify that inhibiting both the upstream and downstream components of PI3K/Akt/mTOR signaling is an effective approach to synergistically decrease cell proliferation.

### Co-treatment of BEZ235 or ZSTK474 with temsirolimus induces G1 cell cycle arrest and up-regulates p27

**Figure 5:**
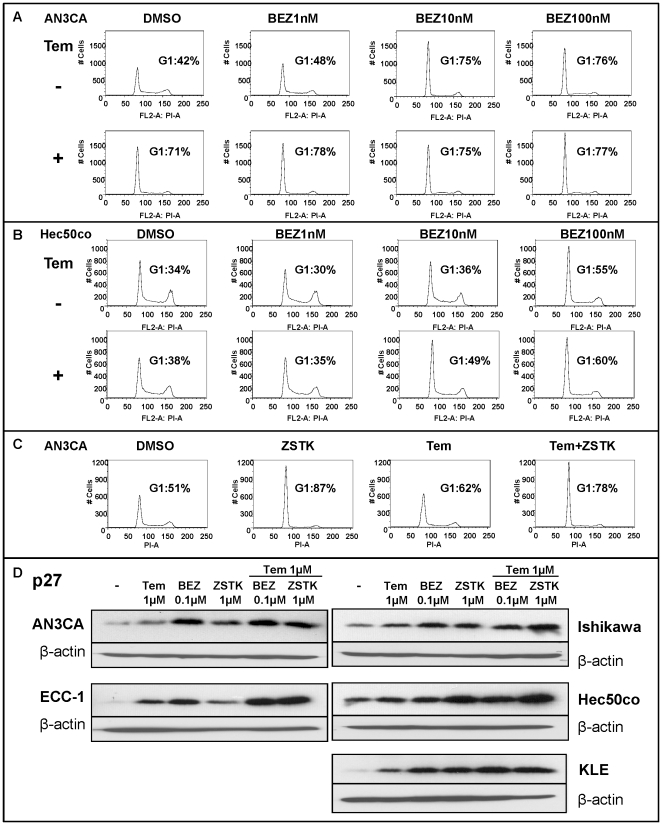
BEZ235 alone or in combination with temsirolimus induces G1 cell cycle arrest and p27 expression. *A–B*, AN3CA (A) or Hec50co (B) cells were treated for 24 hrs or 72 hrs, respectively, with vehicle (DMSO) or BEZ235 (1 nM–100 nM) in the presence or absence of 1 nM temsirolimus. Cell cycle distribution was analyzed by flow cytometry and the percentage of cells in G1 determined. *C*, AN3CA cells were treated for 24 hrs with vehicle (DMSO) or ZSTK474 (1 µM) in the presence or absence of 1 nM temsirolimus. Cells were analyzed as in A. *D*, Expression of the cyclin-dependent kinase inhibitor p27 was assessed by Western blotting 24 hrs after the indicated treatments.

Temsirolimus and BEZ235 have been reported to inhibit cancer cell growth by inducing G0/G1 cell cycle arrest [26], [27]. Since we have two cell populations whose proliferation is differentially affected by temsirolimus treatment (sensitive vs. resistant), we chose two representative cell lines (AN3CA-sensitive; Hec50co-resistant) as models to test the combination approach on cell cycle progression. Cell cycle content analysis of AN3CA cells demonstrated that temsirolimus treatment increased the percent of cells in G1 from 42% (DMSO control) to 71% after only 24 hours (Fig. 5A). Co-treatment with BEZ235 and temsirolimus led to a slight increase of cells in G1 compared to temsirolimus alone at 24 hours (Fig. 5A). In contrast, treatment of Hec50co cells with temsirolimus alone caused a 4% increase in the G1 population, which required 72 hours of prolonged exposure to demonstrate (Fig. 5B). However, when temsirolimus was combined with BEZ235, the G1 population increased to 60% by 72 hours (Fig. 5B). Further, ZSTK474 effects in combination with temsirolimus (Fig. 5C) replicated those of BEZ235 (Fig. 5A), suggesting that G1 arrest is a common method by which combined inhibition of PI3K and mTOR achieves the decrease in cell viability observed in Figure 4. Also of note, the G1 block was achieved rapidly in sensitive cells, but eventually was observed even in resistant cells.

Expression of the cyclin dependent kinase inhibitor, p27, was next tested in order to explore the cell cycle regulatory proteins downstream of mTOR that might explain the G1 block. As shown in Figure 5D, single and combined drug treatments promoted increased expression of p27 at the protein level, implicating induction of p27 in the mechanism of G1 arrest.

### Combined treatment induces autophagy as a mechanism of cell death

**Figure 6:**
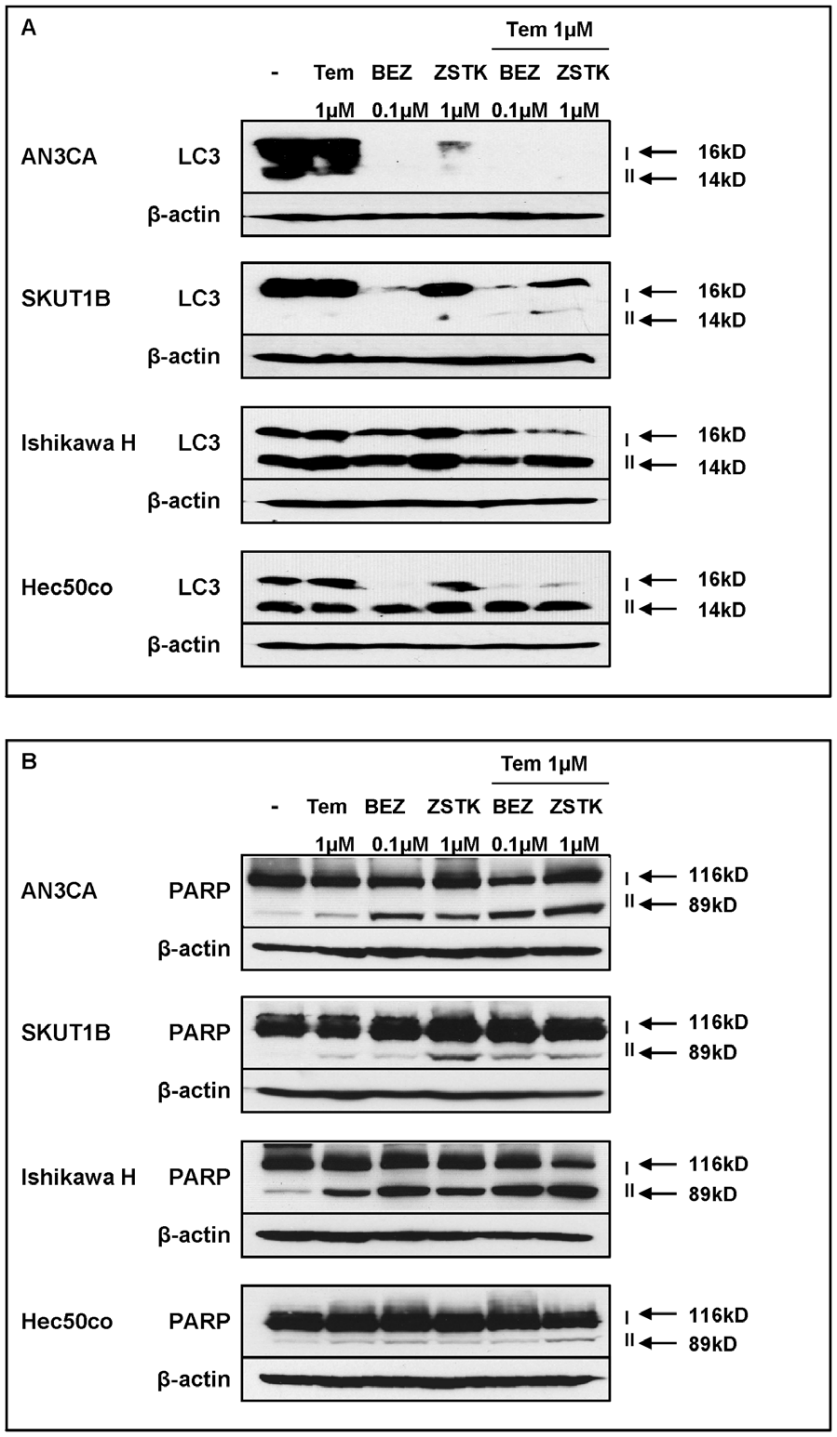
Combination treatment induces autophagy and PARP cleavage. (A) Expression of the autophagy marker LC3 was assessed by Western blotting after the indicated treatments for 24 hrs. Arrows denote full-length LC3-I (16 kDa) and cleaved LC3-II (14 kDa). (B) PARP cleavage was assessed by Western blotting after the indicated treatments for 48 hrs–72 hrs. Arrows denote full-length PARP (116 kDa) and cleaved PARP (89 kDa).

We next characterized the mechanism of cell death in response to combination therapy. Examination of several apoptotic markers, including caspase 3, revealed that the cells did not undergo caspase-dependent apoptosis (data not shown). However, several studies have demonstrated that both down-regulation of Akt [28], [29] and treatment with rapalogs [26] promote autophagy, which can lead to caspase-independent apoptosis characterized by poly (ADP-ribose) polymerase (PARP) cleavage [30], [31]. Therefore, we first tested processing of microtubule-associated protein light chain 3-I (LC3-I), which is a common marker for autophagy [32]. As noted in Figure 6A, under our experimental conditions, no loss of LC3-I occurred upon temsirolimus treatment when compared with control. In contrast, BEZ235 alone dramatically reduced the level of LC3-I in all tested cells. Furthermore, combination of temsirolimus with ZSTK474 reduced levels of LC3-I compared with ZSTK474 alone. The reduction in LC3-I levels was accompanied by the expected increase or maintenance of LC3-II in some cell lines (Ishikawa H and Hec50co), further confirming that the cells are undergoing autophagy (Fig. 6A). Since others have reported that BEZ235 induces caspase-independent apoptosis through PARP cleavage [30], we treated cells for longer periods of time (48 to 72 hrs) and examined PARP cleavage as a marker of apoptotic cell death. In both sensitive and resistant cell lines, we observed PARP cleavage following treatment with BEZ235 or ZSTK474, and addition of temsirolimus did not substantially increase the effect (Fig. 6B). Collectively, these data suggest that the mechanism of cell death involves massive autophagy which proceeds into caspase-independent apoptosis.

### Mechanism of synergy between PI3K and mTOR inhibitors

**Figure 7:**
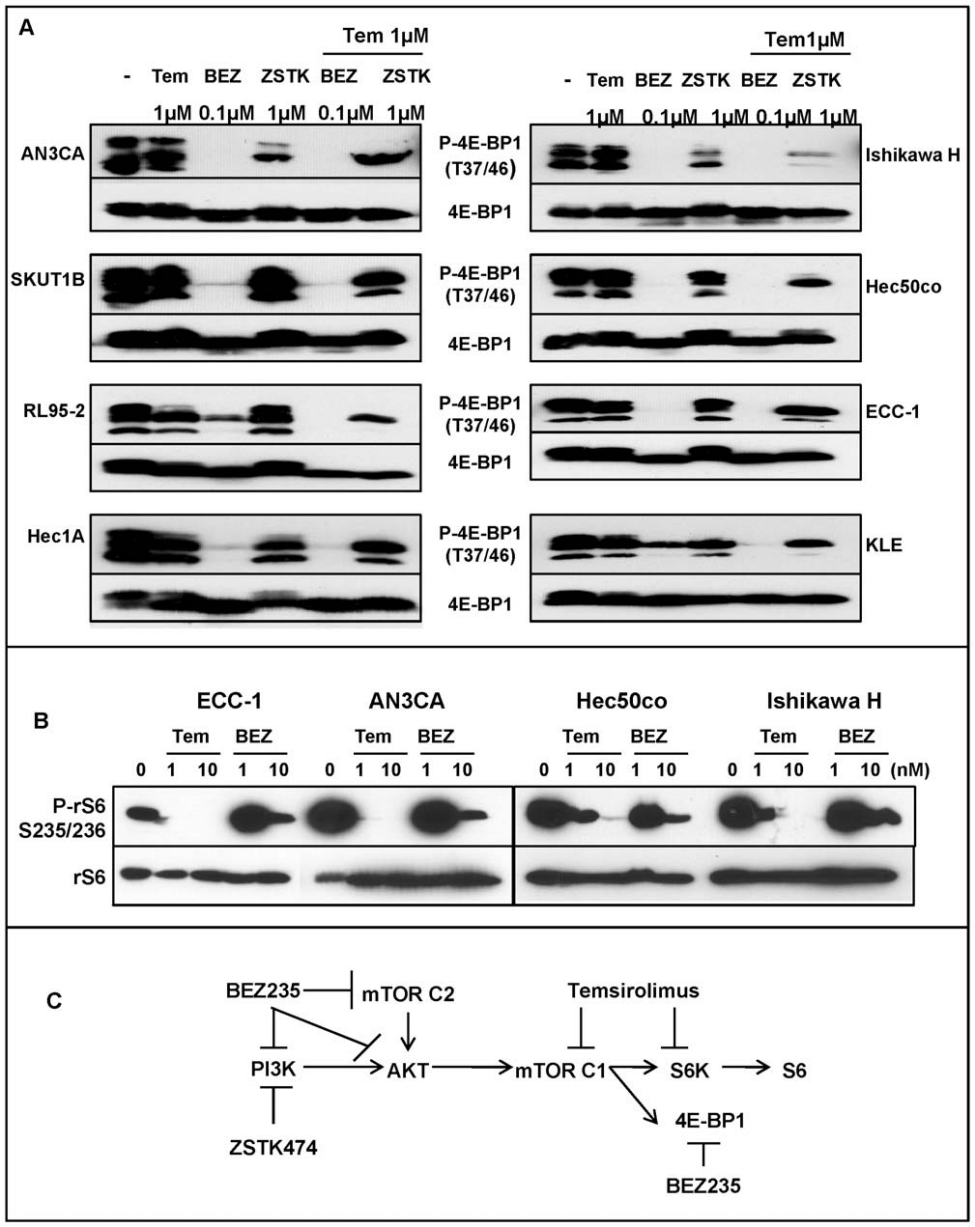
Mechanism of synergistic effect for combination treatment of temsirolimus and BEZ235. *A*, Phosphorylation of 4E-BP1 (P-4E-BP1 T37/T46) was assessed after incubating cells with the indicated treatments for 24 hrs. Total 4E-BP1 expression serves as a loading control. *B*, Cells were treated with 1 nM or 10 nM temsirolimus or BEZ235 for 72 hrs then phosphorylation of rS6 (P-rS6 S235/S236) determined by Western blotting. Total rS6 serves as a loading control. *C*, Proposed mechanism for synergistic effect. Temsirolimus blocks one arm of mTORC1 signaling as evidenced by lack of phosphorylation of rS6. As a dual inhibitor of PI3K and mTOR, BEZ235 acts both upstream as well as downstream of mTORC1 and mTORC2 through inhibition of Akt (upstream) and 4E-BP1 (downstream) phosphorylation. BEZ235 has minimal effects on rS6 phosphorylation, but combination with temsirolimus blocks both arms of mTORC1 signaling. As a specific PI3K inhibitor, ZSTK474 acts upstream of the PI3K/Akt/mTOR signaling pathway, and in combination with temsirolimus, can block rS6 but not 4E-BP1 activation.

Recently, several groups reported that 4E-BP1, not rS6, which are both downstream of mTORC1, is critical for controlling oncogenic Akt signaling [10], [11]. Additionally, it was demonstrated the BEZ235 is more effective at down-regulating 4E-BP1 phosphorylation as compared to rapamycin and second-generation mTOR inhibitors [11]. ZSTK474 targets PI3K, which is upstream of PI3K/Akt/mTOR signaling, while temsirolimus inhibits mTORC1, which is downstream of this pathway. This strategy, targeting both ends of the same pathway, clearly demonstrated synergy in cell proliferation assays (Fig. 4B). However, BEZ235 itself is a dual PI3K/mTOR inhibitor that functions both upstream and downstream of the PI3K/Akt/mTOR pathway and should be sufficient to completely block signaling; nevertheless, a synergistic effect was observed when BEZ235 was combined with another mTOR inhibitor, temsirolimus. This finding was unexpected, and we sought an explanation for why blocking mTORC1 with two different agents produced synergy. We addressed this question by examining the individual effect of BEZ235 and temsirolimus on downstream components of mTORC1 pathway signaling (Fig. 7A, B). The downstream components tested were 4E-BP1 and rS6 (Fig. 7C). Surprisingly, temsirolimus had little or no effect on inhibiting 4E-BP1 phosphorylation compared with control despite its ability to fully block phosphorylation of rS6 (Fig. 7B). On the other hand, BEZ235 completely blocked 4E-BP1 phosphorylation in all tested cell lines but had less effect on rS6 phosphorylation (Fig. 7A, B). When testing the phosphorylation of rS6, we discovered that temsirolimus inhibited it much more effectively (at 1 nM) than BEZ235 at the same concentration (Fig. 7B and Supporting Fig. S3). In summary, to fully block both of the important downstream effectors, both drugs were required. Figure 7C is a schematic model to summarize our findings on the specific mechanistic functions of PI3K/Akt/mTOR inhibitors in the treatment of cancers that are reliant on this pathway.

## Discussion

Temsirolimus is currently in phase II trials for advanced endometrial cancer and has shown some promise. However, lack of initial response to therapy as well as the development of acquired drug resistance continues to be problematic. To more fully understand the therapeutic potential of mTOR inhibition in endometrial cancer, we first examined the effect of temsirolimus alone on the viability of a panel of endometrial cancer cell lines. We sought to distinguish between cellular events which predict for primary resistance as well as those events which are linked to the eventual development of acquired resistance. Consistent with other types of cancer, primary resistance to temsirolimus is found in a subset of these cell lines. Our data suggest that primarily resistant cells lack robust Akt signaling, are unable to phosphorylate Akt at baseline, and express PTEN. In contrast, the most sensitive cell lines have lost PTEN expression and have high baseline phosphorylation of Akt. Our data demonstrate that in these cells, temsirolimus treatment promotes a further increase in Akt phosphorylation, indicating that signaling through the pro-survival PI3K/Akt pathway is likely how these endometrial cancer cell lines eventually circumvent mTOR inhibition. These results are consistent with previous reports in other types of cancers documenting compensatory Akt phosphorylation in response to other rapalogs [7], [16], [24]. This has been observed in xenograft models of lung cancer [24] as well as in advanced colon and breast cancer tissues following rapalog therapy [16]. The elevated Akt phosphorylation is thought to be a predominant driving force in resistance to temsirolimus treatment in these cancers [7].

To overcome resistance, we adopted a combination strategy. Dual treatment with temsirolimus and the PI3K inhibitor ZSTK474 or the PI3K/mTOR inhibitor BEZ235 overcame the temsirolimus-induced Akt hyper-phosphorylation, which is a marker for developing acquired resistance; furthermore, this treatment strategy synergistically decreased viability and promoted G1 cell cycle arrest even in the cell lines that were primarily resistant to temsirolimus alone. These findings are consistent with a recent study in melanoma cells in which dual treatment with the PI3K inhibitor PI-103 and rapamycin reversed compensatory Akt phosphorylation and induced cell cycle arrest, and xenograft studies demonstrated reduced tumor growth with this combination strategy [33]. We extend these findings herein to define a potential mechanism by which the combination therapy promotes cell death.

We found that BEZ235 alone blocked PI3K, mTORC1, and mTORC2 activity, in particular 4E-BP1 phosphorylation at a dose of 100 nM. However, BEZ235 was less effective in blocking rS6 phosphorylation. In comparison, temsirolimus completely abrogated phosphorylation of rS6 at 1 nM. Thus, combining both agents (BEZ235 and temsirolimus) completely inhibited signaling throughout the pathway and synergistically induced cell death.

Currently, combinatorial therapies are being applied to prevent resistance to single-agent treatments such as rapalogs. Examples of targeted small-molecule inhibitors under investigation include BEZ235 (dual PI3K/mTOR inhibitor) [27], AZD2171 (dual VEGF2/PDGFR inhibitor); LBH589 (histone deacetylase inhibitor) [23], LY294002 (PI3K inhibitor) [24], AZD6244 (MEK inhibitor) [25], and ZSTK474 (PI3K inhibitor) [34]. BEZ235 is a novel orally bioavailable inhibitor originally designed as a pan-PI3K family inhibitor based on the p110γ (catalytic subunit of PI3K) kinase domain structure [6], [27]. Interestingly, when this compound was evaluated in preclinical studies, *in vitro* kinase assays revealed it also targets mTOR at a concentration of 20.7 nM [27]. Therefore, BEZ235 is classified as a dual inhibitor that is capable of targeting both upstream (PI3K) and downstream (mTORC1/mTORC2) of the PI3K/Akt/mTOR axis. BEZ235 has been reported to inhibit growth and proliferation and induce apoptosis in a variety of tumor cell lines [27], [35], including breast cancer cells with mutant or amplified *PIK3CA* [30]. BEZ235 showed antitumor activity in nude mice with few side effects [27]. A recent report from a phase I study of BEZ235 in 59 patients with advanced solid tumors demonstrated antitumor effects and a favorable safety profile [36]. ZSTK474, a pan-class I PI3K inhibitor, also demonstrated high potency against a panel of cancer cell lines and human tumor xenografts without toxicity to major organs [34], [37]. As discussed above, among all drugs tested, the agents which produced synergy with temsirolimus in our models were BEZ235 and ZSTK474.

A main conclusion of our study is that combination treatment of ZSTK474 or BEZ235 with temsirolimus synergizes to decrease viability in endometrial cancer cell lines. A potential mechanism of synergy from co-treatment with ZSTK474 and temsirolimus is the vertical blockade of hyper-activated PI3K/Akt/mTOR signaling, specifically the simultaneous targeting of the upstream component PI3K by ZSTK474 and the downstream component mTOR (p70S6K) by temsirolimus. Temsirolimus alone only blocks rS6K activity downstream of mTORC1, whereas signaling through the other mTORC1 target 4E-BP1 is left intact. It has been documented in the literature that signaling through 4E-BP1 is required for Akt-mediated oncogenesis [10]; therefore, inhibition of all components of this pathway is necessary to prevent tumor growth. Our data indicate that, in addition to inhibition of Akt activation, BEZ235 effectively blocks this residual signaling through 4E-BP1, which, when combined with temsirolimus inhibition of rS6K, synergistically blocks all arms of the PI3K/Akt/mTOR pathway. Besides the observed inhibition of 4E-BP1 and rS6 with combined BEZ235 (dual PI3K/mTOR inhibitor) and temsirolimus (mTORC1 inhibitor), another possibility might explain the observed synergy. Temsirolimus and BEZ235 target different structural domains of mTOR: temsirolimus is an allosteric inhibitor that targets the FKBP12-rapamycin-binding (FRB) domain while BEZ235 is a catalytic inhibitor that targets the kinase domain. The inhibitory potential of targeting two structurally distinct regions of the same protein may, therefore, contribute to the synergistic effect we observed when cells were treated with temsirolimus and BEZ235 compared to single agent treatment alone.

It has previously been shown that treatment with BEZ235 or ZSTK474 results in cell cycle arrest at G1 [27], [37]. Our study demonstrates that cells were more likely to arrest in G1 if they had been treated with either BEZ235 or ZSTK474 with temsirolimus compared to controls or single agent treatment. This may be attributed to the ability of BEZ235 to promote increased expression of the CDKI p27 [27]. Accordingly, we also detected elevated p27 expression when endometrial cancer cells were treated with BEZ235 alone or in combination with temsirolimus. While inhibition of prosurvival Akt signaling is cytotoxic, the mechanism of cell death involves autophagy and apoptosis [26]. We observed a decrease in the autophagy marker, LC-3I, in response to dual mTOR/PI3K inhibition, implicating autophagy. Others have shown that depletion of all three Akt isoforms promoted tumor regression through initiation of autophagy [29], and inhibition of mTOR with the alkylphospholipid perifosine induces autophagic cell death [28]. BEZ235 has also been shown to induce caspase-independent apoptosis in a mechanism that includes PARP cleavage [30], which we also observed in our study. Taken together, these data suggest that the mechanism of cell death is through autophagy and caspase-independent apoptosis.

Molecular profiling of the endometrial cancer cell lines revealed that sensitivity toward drug treatment correlates with loss of PTEN expression and hyper-activation of Akt. In endometrial tumors, loss of PTEN has previously been shown to correlate with elevated Akt phosphorylation and results in poor outcomes [17], [38]. Our findings are consistent with earlier studies showing low expression of PTEN in RL95-2, AN3CA, ECC-1, and Ishikawa H cells and high expression of PTEN in Hec50, Hec1A, and KLE cells [4], [39]. Furthermore, RL95-2, AN3CA, ECC-1, and Ishikawa H cells harbor mutant PTEN, whereas KLE, Hec50, Hec1A, and Hec1B (a substrain of Hec1A) cells express wildtype PTEN [40], [41]. The fact that Hec50 cells contain both wildtype PTEN and high Akt phosphorylation can be explained by recent data demonstrating that *PI3KR1*, a regulatory subunit of PI3K, is mutated in Hec50 cells and thus may phenocopy loss of PTEN [4]. Additional investigation is necessary to understand why Ishikawa H cells, which have high Akt phosphorylation and a loss of active PTEN, are relatively resistant to temsirolimus. These data highlight the fact that molecular profiling does not always predict for response due to the complexity of pathways governing tumor initiation and progression. Furthermore, basal Akt phosphorylation correlates with response to another rapalog, RAD001, in a panel of various cancer cell lines [8]. Studies involving PTEN^+/−^ mice or cell lines devoid of PTEN show that PTEN-deficient tumors are sensitive to mTOR inhibition [20]. A phase I clinical trial demonstrated that 63% of patients with PTEN-negative tumors displayed tumor regression when treated with drugs targeting the PI3K/Akt/mTOR signaling pathway [42]. These results are consistent with our data that cell lines with little or no PTEN are more sensitive to temsirolimus alone and with the combination of temsirolimus and BEZ235. In accord with our findings, single-agent BEZ235 has been shown to inhibit proliferation of endometrial cancer cells harboring *PIK3CA* and/or *PTEN* mutations by other investigators [43]. These studies reported herein promote a further understanding of the potential underlying mechanisms of both primary cell resistance and the development of acquired resistance after therapy, both of which can be overcome with the combination of mTOR and PI3K inhibitors. Our data underscore the need to inhibit PI3K/Akt/mTOR signaling at multiple levels to achieve sustained cellular responses. These data will enhance the rational use of combinatorial regimens involving temsirolimus and PI3K inhibitors in future clinical trials.

## Supporting Information

Figure S1 Efficacy of temsirolimus in a panel of endometrial cancer cell lines. The indicated cell lines were treated with vehicle or 1 µM temsirolimus for 24 hrs. Lysates were obtained and equal amounts Western blotted for phospho-mTOR, total mTOR, phospho-rS6, or total r6S.
(TIF)

Figure S2 Effect of various combination therapies on Akt phosphorylation. Ishikawa H (upper panels) or Hec50co (lower panels) cells were treated with temsirolimus in the presence or absence of the indicated molecular inhibitors for 24 hrs at the noted concentrations. Lysates were obtained and equal amounts Western blotted for phospho-Akt or total Akt.
(TIF)

Figure S3 Effect of BEZ235, ZSTK474, and temsirolimus on rS6 phosphorylation. Phosphorylation of rS6 (P-rS6 S235/236) was assessed after incubating cells with the indicated treatments for 24 hrs. Total rS6 expression serves as a loading control.
(TIF)

Table S1 Temsirolimus and BEZ235 IC_50_ and combination index (CI) for combined temsirolimus and BEZ235 treatment in the panel of eight endometrial cancer cell lines.
(TIF)

Table S2 Panel of molecular inhibitors explored for combination therapy with temsirolimus.
(TIF)
