# Supplementary figures and images for: A Mechanism for Synergy with Combined mTOR and PI3 Kinase Inhibitors
Source: PLoS One. 2011 Oct 19;6(10):e26343. doi: 10.1371/journal.pone.0026343 (PMC3198385; doi:10.1371/journal.pone.0026343)

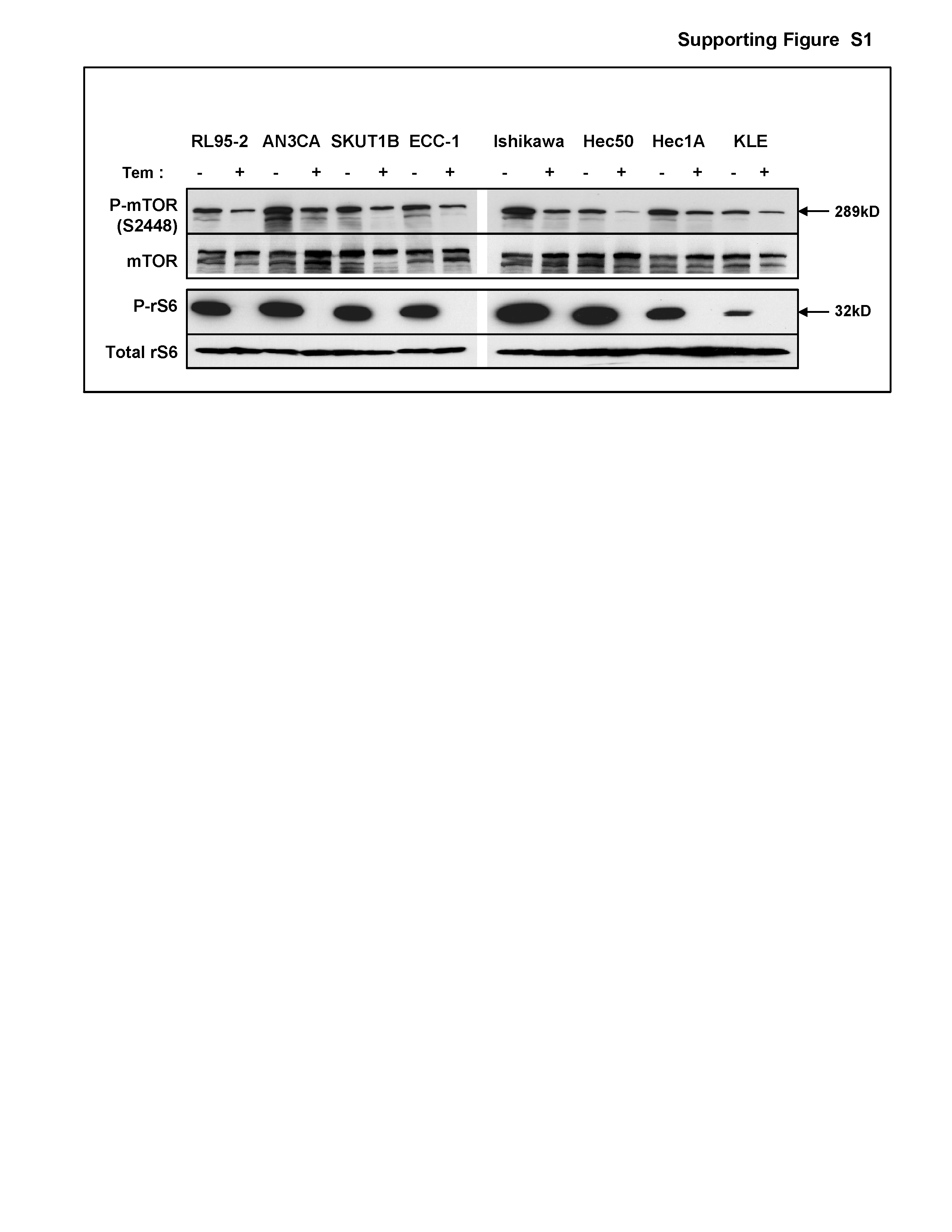

Supplement: Figure S1 — Efficacy of temsirolimus in a panel of endometrial cancer cell lines. The indicated cell lines were treated with vehicle or 1 µM temsirolimus for 24 hrs. Lysates were obtained and equal amounts Western blotted for phospho-mTOR, total mTOR, phospho-rS6, or total r6S. (TIF) [file pone.0026343.s001.tif]

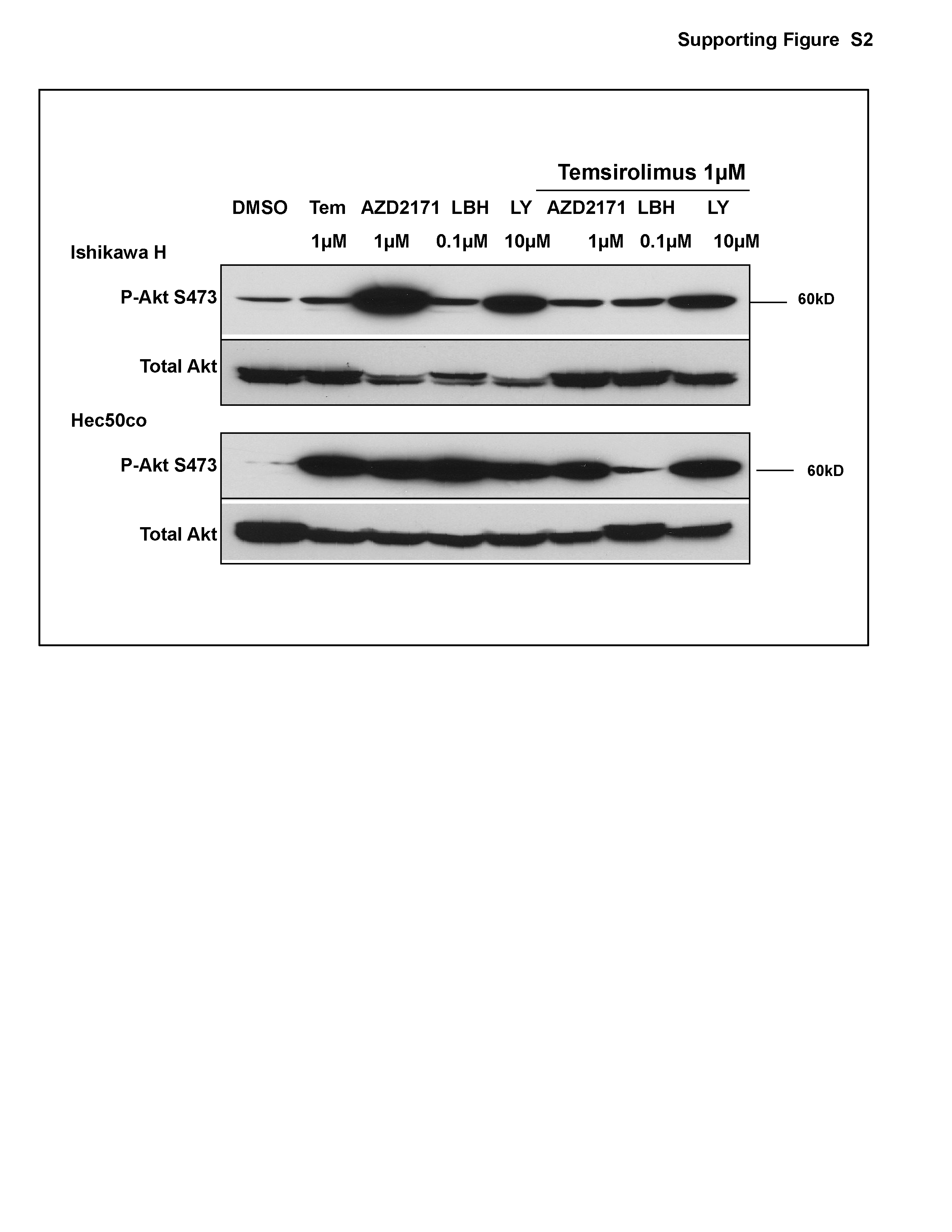

Supplement: Figure S2 — Effect of various combination therapies on Akt phosphorylation. Ishikawa H (upper panels) or Hec50co (lower panels) cells were treated with temsirolimus in the presence or absence of the indicated molecular inhibitors for 24 hrs at the noted concentrations. Lysates were obtained and equal amounts Western blotted for phospho-Akt or total Akt. (TIF) [file pone.0026343.s002.tif]

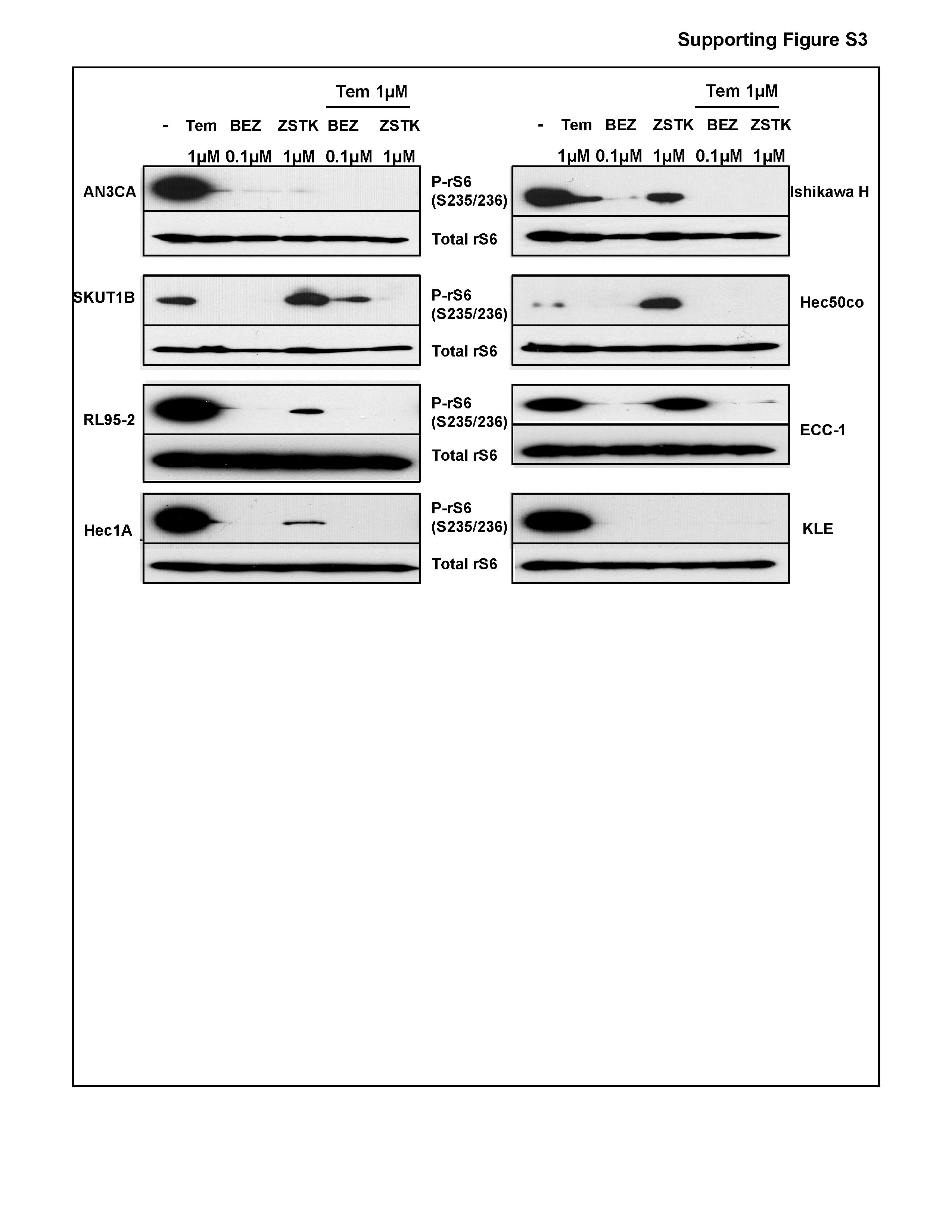

Supplement: Figure S3 — Effect of BEZ235, ZSTK474, and temsirolimus on rS6 phosphorylation. Phosphorylation of rS6 (P-rS6 S235/236) was assessed after incubating cells with the indicated treatments for 24 hrs. Total rS6 expression serves as a loading control. (TIF) [file pone.0026343.s003.tif]

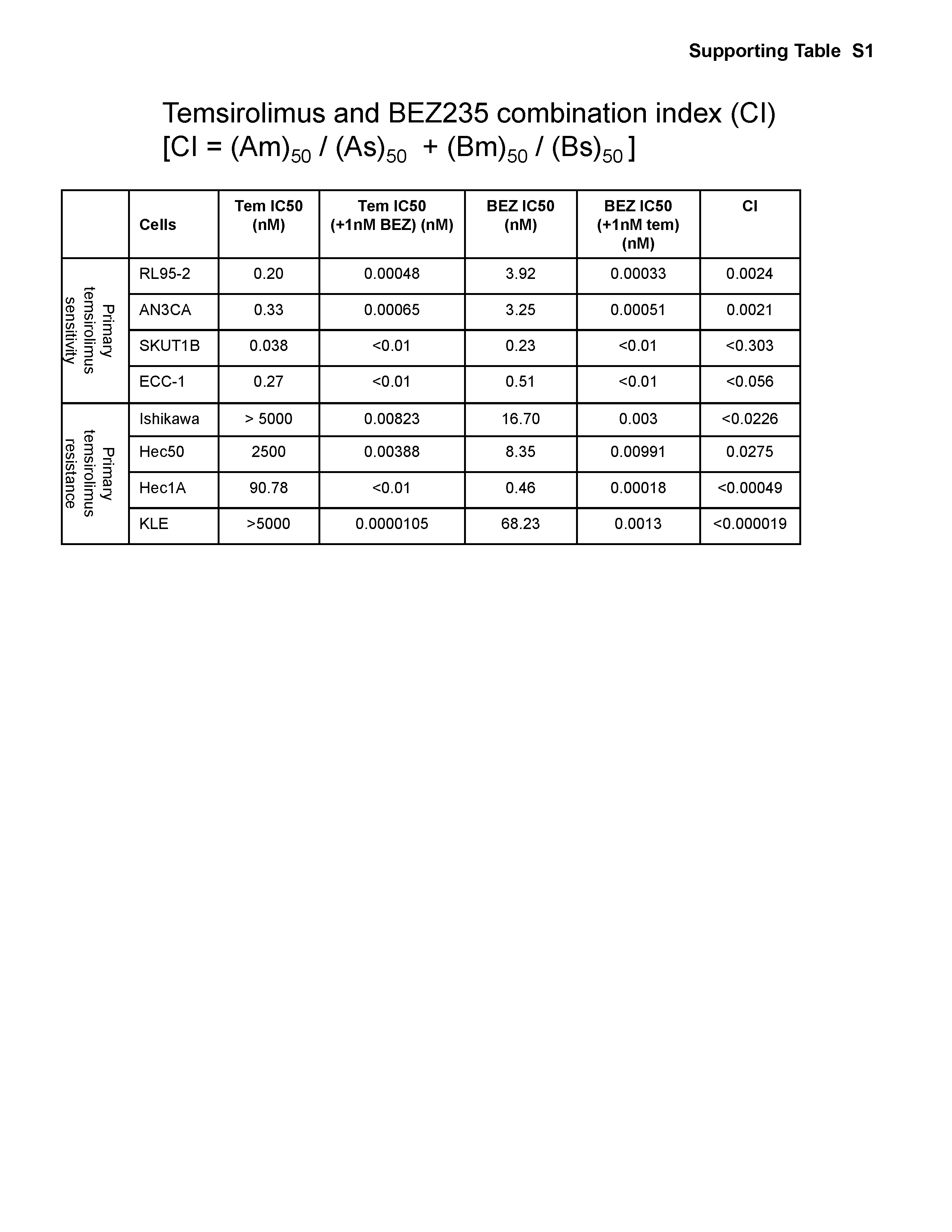

Supplement: Table S1 — Temsirolimus and BEZ235 IC50 and combination index (CI) for combined temsirolimus and BEZ235 treatment in the panel of eight endometrial cancer cell lines. (TIF) [file pone.0026343.s004.tif]

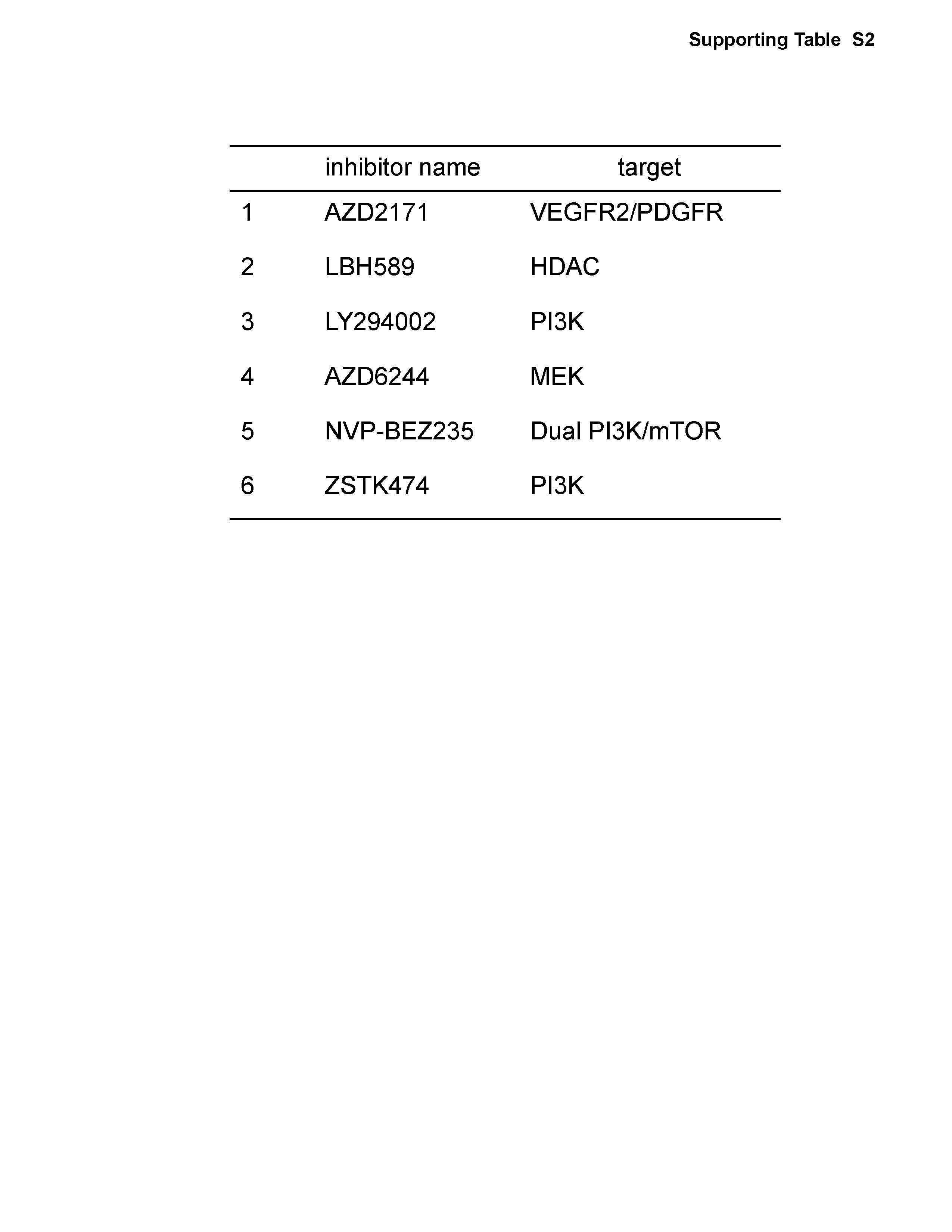

Supplement: Table S2 — Panel of molecular inhibitors explored for combination therapy with temsirolimus. (TIF) [file pone.0026343.s005.tif]
